# Supplementary material for: Novel Driver Strength Index highlights important cancer genes in TCGA PanCanAtlas patients
Source: PeerJ. 2022 Aug 11;10:e13860. doi: 10.7717/peerj.13860 (PMC9375969; doi:10.7717/peerj.13860)
Supplement: Supplemental Information 2 [file peerj-10-13860-s002.zip › COHORTS/genes plots/age/2021_8_16_14_9_distribution_cohorts_males_60-64.pdf]

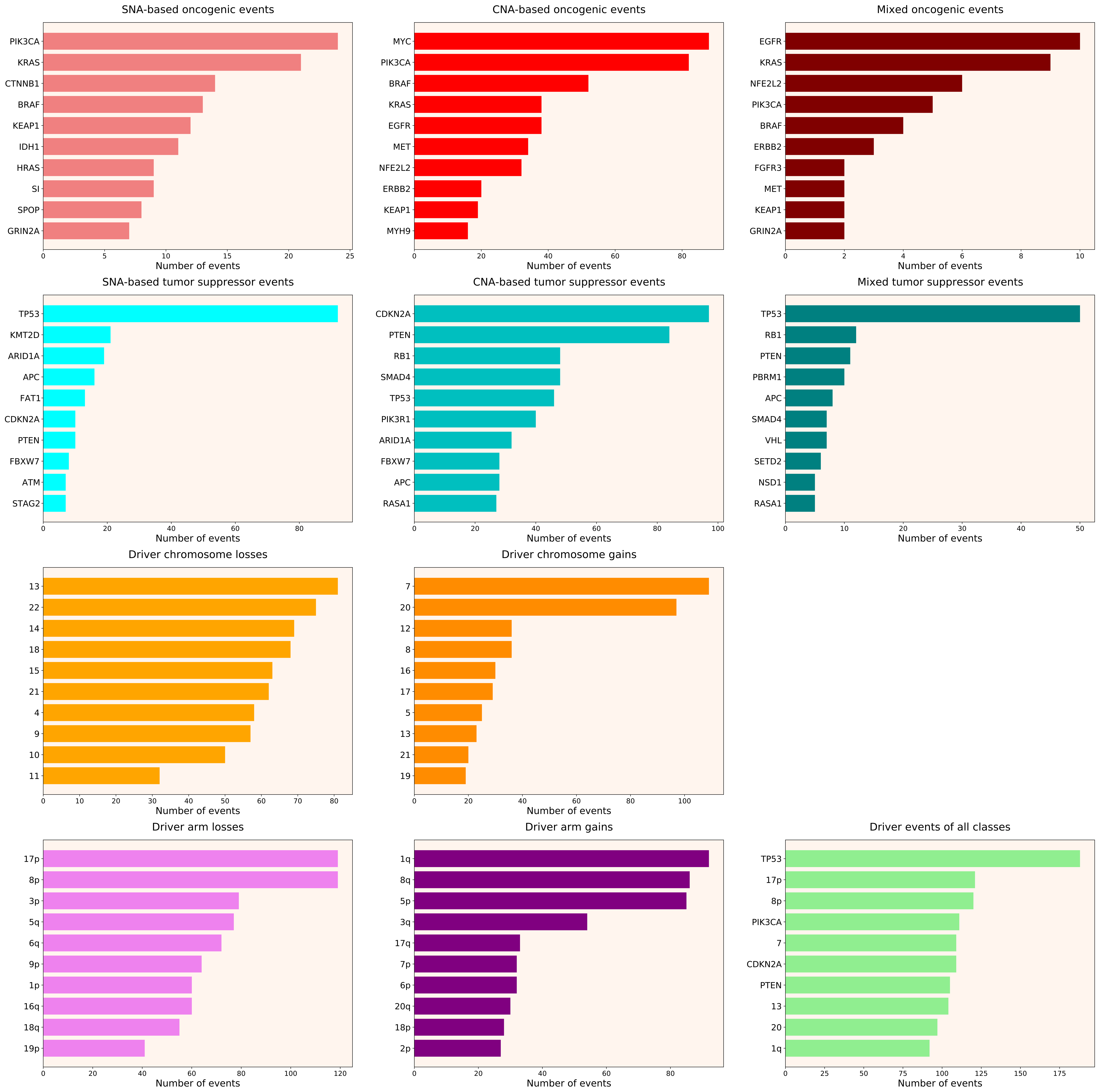
SNA-based oncogenic events
PIK3CA
KRAS
CTNNB1
BRAF
KEAP1
IDH1
HRAS
SI
SPOP
GRIN2A
Number of events
CNA-based oncogenic events
MYC
PIK3CA
BRAF
KRAS
EGFR
MET
NFE2L2
ERBB2
KEAP1
MYH9
Number of events
Mixed oncogenic events
EGFR
KRAS
NFE2L2
PIK3CA
BRAF
ERBB2
FGFR3
MET
KEAP1
GRIN2A
Number of events
SNA-based tumor suppressor events
TP53
KMT2D
ARID1A
APC
FAT1
CDKN2A
PTEN
FBXW7
ATM
STAG2
Number of events
CNA-based tumor suppressor events
CDKN2A
PTEN
RB1
SMAD4
TP53
PIK3R1
ARID1A
FBXW7
APC
RASA1
Number of events
Mixed tumor suppressor events
TP53
RB1
PTEN
PBRM1
APC
SMAD4
VHL
SETD2
NSD1
RASA1
Number of events
Driver chromosome losses
13
22
14
18
15
21
4
9
10
11
Number of events
Driver chromosome gains
7
20
12
8
16
17
5
13
21
19
Number of events
Driver arm losses
17p
8p
3p
5q
6q
9p
1p
16q
18q
19p
Number of events
Driver arm gains
1q
8q
5p
3q
17q
7p
6p
20q
18p
2p
Number of events
Driver events of all classes
TP53
17p
8p
PIK3CA
7
CDKN2A
PTEN
13
20
1q
Number of events
